# Passive microinjection within high-throughput microfluidics for controlled actuation of droplets and cells

**DOI:** 10.1038/s41598-019-43056-2

**Published:** 2019-04-30

**Authors:** Milad Azarmanesh, Morteza Dejam, Pooya Azizian, Gurkan Yesiloz, Abdulmajeed A. Mohamad, Amir Sanati-Nezhad

**Affiliations:** 1 Department of Mechanical and Manufacturing Engineering, University of Calgary, Calgary, Alberta T2N 1N4 Canada; 2 Center for Bioengineering Research and Education, University of Calgary, Calgary, Alberta T2N 1N4 Canada; 3 Department of Petroleum Engineering, College of Engineering and Applied Science, University of Wyoming, 1000 E. University Avenue, Laramie, Wyoming 82071-2000 USA; 4 Department of Mechanical Engineering, Babol Noshirvani University of Technology, Shariati St., Babol, 4714871167 Iran

**Keywords:** Protein delivery, Mechanical engineering, Computational science

## Abstract

Microinjection is an effective actuation technique used for precise delivery of molecules and cells into droplets or controlled delivery of genes, molecules, proteins, and viruses into single cells. Several microinjection techniques have been developed for actuating droplets and cells. However, they are still time-consuming, have shown limited success, and are not compatible with the needs of high-throughput (HT) serial microinjection. We present a new passive microinjection technique relying on pressure-driven fluid flow and pulsative flow patterns within an HT droplet microfluidic system to produce serial droplets and manage rapid and highly controlled microinjection into droplets. A microneedle is secured within the injection station to confine droplets during the microinjection. The confinement of droplets on the injection station prevents their movement or deformation during the injection process. Three-dimensional (3D) computational analysis is developed and validated to model the dynamics of multiphase flows during the emulsion generation. We investigate the influence of pulsative flows, microneedle parameters and synchronization on the efficacy of microinjection. Finally, the feasibility of implementing our microinjection model is examined experimentally. This technique can be used for tissue engineering, cells actuation and drug discovery as well as developing new strategies for drug delivery.

## Introduction

Encapsulation of drug molecules and species like viruses inside a single cell, or delivery of molecules and cells into droplets has led to the creation of compound structures^1–4^, controlled chemical reactions^1,5^, development of drugs for evolving cells and enzymes^6^, and regulation of cell fates. The delivery of particles into droplets have been established using double emulsion techniques such as hierarchical T-junction^7–11^, flow-focusing^2,8,9,12,13^, co-flowing^14,15^, K-channel^16^ and cross-flowing^4,17^. Droplet microfluidics is used for capturing and indexing thousands of individual cells in nano-liter droplets^18^, suitable for small *in vivo* clinical samples like tumors and tissue micro biopsies^19–21^. Droplet microfluidics has also been combined with electroporation and magnetic tweezers to label cells remotely using weak external magnetic fields applicable for high-throughput gene transfection^22^. Traditional microinjection techniques have been used for DNA-based crop improvement^23–25^ and altering the cell fate^26,27^. These techniques are based on active methods such as valves^28^, moving injector^29^ and rotating injector^30^. However, they are costly and time-consuming, demand experienced operators, suffer from low productivity and success rate, and are not compatible with the needs of high-throughput microinjectors.

Automated microinjection techniques have been developed to increase speed^29^ and accuracy^30,31^ of microinjection. Sun and his colleagues^32,33^ presented an autonomous microrobotic system by a pipette holder and an injector to achieve a high injection success rate. However, the injection needed to be conducted under the supervision of experts^34^. Abate *et al*.^35^ presented new droplet pico-injectors that trigger the droplets by an electric field within microfluidics with high-throughput (HT) performance of sub-picoliter precision. They used pressurized channels to control the volume of reagents delivered into individual droplets^36^. This method was employed by others to destabilize droplets interface^37,38^ and facilitate injection of reagents into droplets^37,39^. Adamo and Jensen^28^ developed an automated microinjector under which the cell is steered toward the fixed microneedle via a passive fluid flow for the delivery of particles and positioned by pressurized air to keep the particle in place. This system uses two integrated valves one to supply the pressure needed for piercing the cells and the other to lift the cell off the microneedle via backpressure. However, the injection still lasts about 0.1 seconds. Despite advances in automated microinjections, the present techniques have limitations either in accuracy or speed of injection. Also, the injection of an immiscible phase into droplets or cells is, however, challenging due to the opening of a considerable area of the droplet interface or cell membrane. Microinjection techniques have not yet been considered for injection of immiscible materials into micro-droplets due to the complexity of the injection process, slow production rate, immiscible conditions regarding the multi interfaces and active structures. Moreover, a comprehensive investigation of the dynamics of immiscible fluid motions and droplet actuation during the microinjection process is not yet revealed.

The success of passive microinjection within microfluidics is heavily dependent on parameters adjustment which necessitates the development of a 3D numerical model for the accurate control of flow and injection parameters. Analytical and numerical methods have been developed to model the physics of double emulsion formation^2,10,14,40^. We previously developed a three-dimensional (3D) model to simulate a hierarchical T-junction microchannel for creating the structure of double emulsion^2,41^, double-component double emulsion, and viscoelastic double emulsion^10^. However, due to the complexity of the 3D numerical model and the needs to adjust a large number of parameters, there is no report on modeling the passive microinjection of an immiscible fluid into droplets formed within microfluidics.

This work presents a passive and automated microinjection technique and employs a pressure-driven fluid flow system for the high-throughput transfer of droplets to the injection station and the successful piercing and high-speed delivery of immiscible liquids/solids into droplets via an embedded injector. This 3D computational model investigates the effect of several multiphase pulsating flows on the interaction of three immiscible fluids. Thanks to the simulation optimization, this passive microinjection technique significantly improved the accuracy and speed of injection. Compared to active microinjection techniques with the injection cycle period of above 0.1 s, our technique reduced the injection cycle down to approximately 3 ms with a minimal error in the delivery into droplets. Our proposed microinjection method is highly feasible to implement given the success of previous experimental works in synchronizing the flow of multiple streams within microchannels with pico-liter precision^35^. The passive microinjection technique can provide much cheaper and higher efficient delivery of genes, small molecules, proteins and viruses into single cells, or precise non-invasive introduction of cells and particles into droplets.

## Results

**Figure 1:**
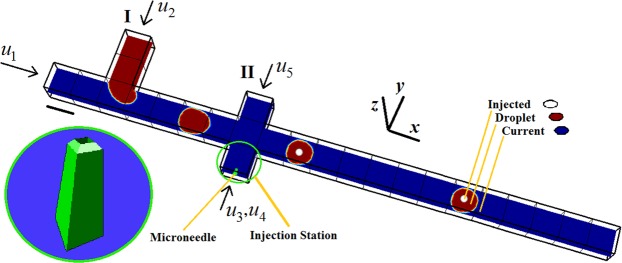
Schematic of the passive and automated microinjection within a microfluidic device. The inlet flows are shown with arrows. The inlets 1 to 5 are related to the inlets for the main channel, T-junction, microneedle, injection station and the top part of the cross-junction, respectively. The microneedle is magnified. The red droplet is named as Droplet. The small white droplet is named as Injected. The blue phase is named as Current. The scale bar is 50 $\mu m$.

Three different phases, named as Droplet, Current, and Injected, interact with each other to control the microinjection into flowing droplets. The design and structure of our proposed microinjection system are shown in Fig. 1. Following the formation of Droplet at the T-junction, it moves to the cross-junction, fits the injection station, and is filled with the Injected phase. The double emulsion is defined as the Droplet phase containing Injected phase inside. The double emulsion is then gently marched to the downstream microchannel (Supplementary Video SV1). The passive scenario of microinjection begins with the interaction of the Current and Droplet phases at the T-junction, where the dripping instability separates the Droplet phase to droplets marching them downstream with a defined size and immutable distance from each other^10^. There are five possible droplet formation regimes of Squeezing, Transition, Dripping, Jetting and parallel due to the instability at the T-junction, which manages the duration of each injection cycle and period of droplet formation. The flow rate ratio of Current to Droplet and the geometry of T-junction is meticulously modified to achieve the desired Droplet size and period of droplet formation. In this work, the dripping regime is an appropriate choice for droplet formation at the T-junction because it creates droplets with approximately as equal droplet size as the microchannel width and is compatible with the size of injection station (approximately 1.2–1.7 L/W). Droplets that form the outer sheath of the double emulsions receive an injection from the microneedle. The microinjection process is performed on the droplet while it is confined by the walls of the injection station. The bottom side holds the inlet for both microneedle and pulsating flow of the injection station. The distributed fluid force produces the force needed for the piercing step. The viscosity of Current, Droplet and Injected are set to 2 × 10^−3^ Pa.s, 10^−3^ Pa.s and 1.243 × 10^−3^ Pa.s, respectively. Moreover, their densities are set to 10^3^ kg m^−3^, 1.1 × 10^3^ kg m^−3^ and 2.614 × 10^3^ kg m^−3^. The surface tension between the Current-Droplet and Current-Injected are set to 0.5 × 10^−2^ N m^−1^ and 4 × 10^−2^ N m^−1^, respectively.

**Figure 2:**
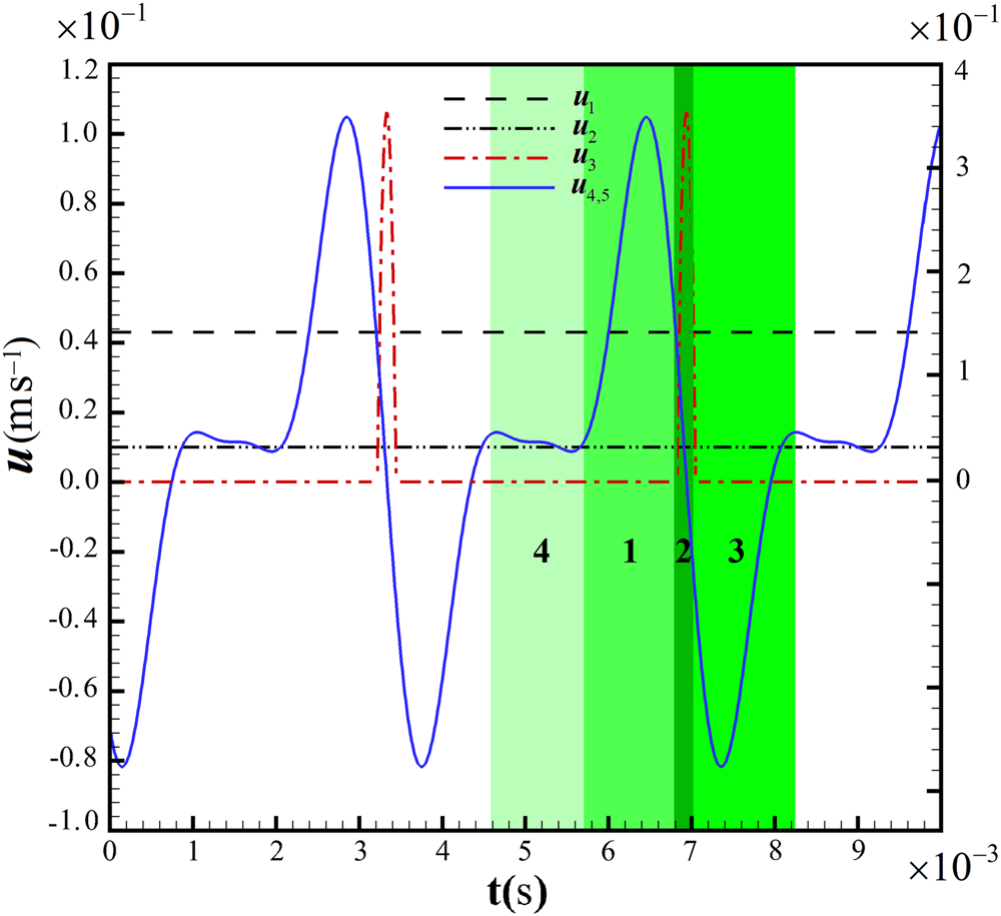
The constant flow rate and pulsating flow patterns for the passive microinjection system. The left-hand axis is related to Droplet and Current (***u***_1_, ***u***_2_ and ***u***_5_). The right-hand axis is related to Injected (***u***_3_). The highlighted areas show the microinjection steps for a single cycle of microinjection started from pushing (1), injection (2), pulling (3) and termination (4). The velocity ***u***_4_ has the same flow pattern as ***u***_5_ with different amplitudes, ***u***_5_ = 2 ***u***_4_.

**Figure 3:**
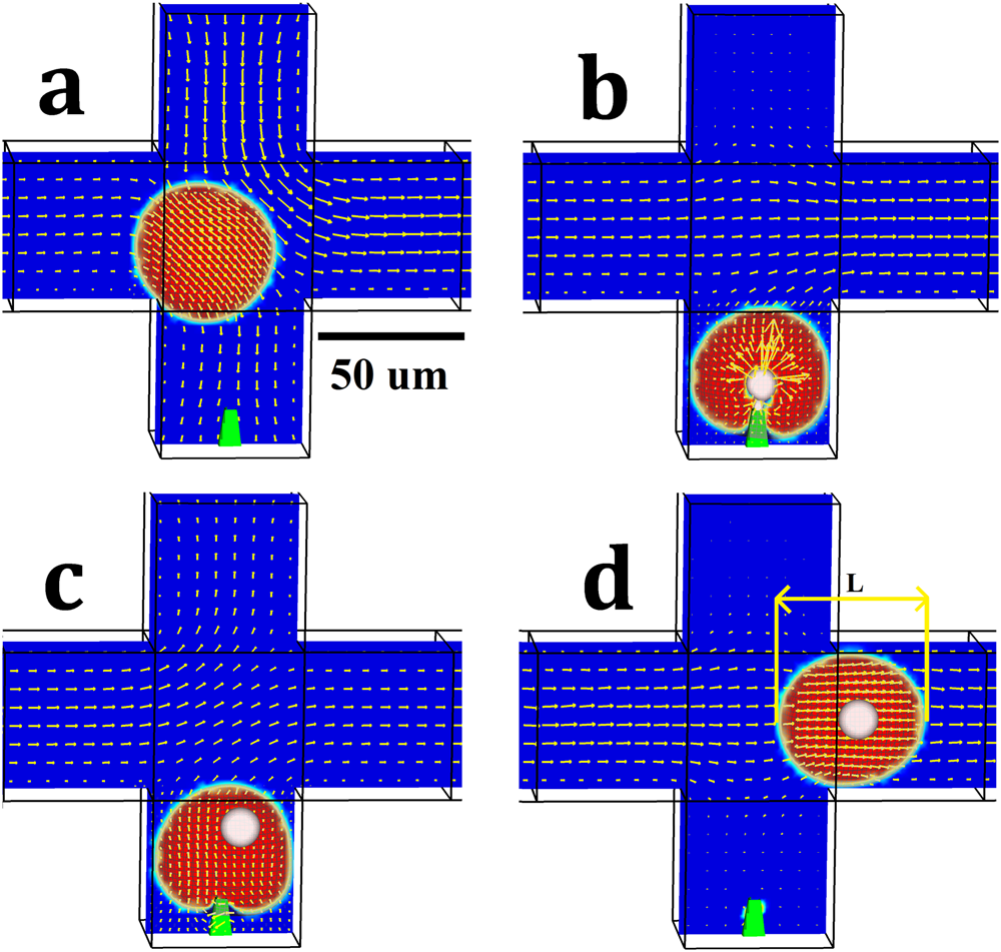
Four steps of the passive microinjection process. (**a**) Pushing t = 6 × 10^−3^ s, (**b**) Resting t = 6.9 × 10^−3^ s, (**c**) Pulling t = 7.1 × 10^−3^ s, (**d**) Moving t = 8 × 10^−3^ s. L denotes the double emulsion length. The scale bar is 50 $\mu m$.

To create double emulsions, two harmonized pulsating flows are introduced, one at the injection station ***u***_4_ and the other one at the cross-junction ***u***_5_ = 2 ***u***_4_ (Fig. 2). They steer Droplet during the microinjection process and conduct it into the injection station where the fixed microneedle is placed (Fig. 3a). The Droplet then remains stationary during the resting step; meanwhile, the microneedle pierces Droplet and delivers Injected (Fig. 3b). The period of injection during the velocity of ***u***_3_ is synchronized with the flow patterns of both the injection station and the top part of the cross-junction to produce double emulsions with Injected confined inside Droplet. Subsequently, the fluid flows are reversed to provide sufficient backpressure to pull the double emulsion off the microneedle. The success of this passive microinjection protocol is dependent on the success in providing high precision and synchronized fluid flows. (Fig. 3c). The net fluxes of the injection station and the cross-junction are finally ceased, and the double emulsion is accordingly departed toward the downstream via the momentum of Current (Fig. 3d). Thus, the double emulsion is produced by a four-step process each of which is highlighted in Fig. 2, the conduction of the Droplet to the injection station; the resting state for injecting into Droplet; pulling the double emulsion off the microneedle; and guiding the double emulsion to the downstream termination by the Current flow. The microinjection cycle is repeated to produce HT protocols for the generation of double emulsions containing Injected inside many Droplets.

Given the fully laminar flow in the microfluidic system, the variation of fluid flows because of pulsating flow patterns does not create vortices. However, the apparent vortices inside the double emulsion provide evidence for fluid circulation and mixing because of the shear stress exerted from Current on Droplet and the variation of laminar fluid flow during the interaction of Droplet with the injection station (Fig. S1a). The vortices also remain active inside Droplet during the resting step. The momentum of Injected also moves the fluid inside Droplet and creates two intense vortices around Injected (Figs S4b, S1c). The vortices have vanished when the injection is stopped and inertial force of Injected moves Droplet off the microneedle (Fig. S1d). The pulsating flow patterns are designed accurately such that the fluid flow is reversed after ceasing the injection. The laminar flow field remains dominant at the downstream of the cycle (Fig. S1e). The generated double emulsion is detached from the microneedle, and the moving step steers the double emulsion towards downstream under completely laminar flow (Fig. S1f).

The first structure of the pulsating flow is provided in Fig. 2. The syringe pump fluctuation pattern is defined as infuse/withdraw autofill cycling profile. However, the fluid inside the syringe does not accordingly sense the same sharp step variation of flow. Therefore, Fourier series is employed to remove the abrupt change of jump in the velocity amplitude and make the flow patterns smoother (Fig. S2b)^42^. The equation $$f(x)={a}_{0}+$$$$\sum _{n=1}^{\infty }({a}_{n}\,\cos \,nx+{b}_{n}\,\sin \,nx)$$ was obtained from the preliminary step flow patterns in Fig. S2a which leads to $${{\boldsymbol{u}}}_{3}=\,\cos \,t+\,\cos \,2t\,{\rm{and}}\,{{\boldsymbol{u}}}_{5}=\,\sin \,t-\,\sin \,2t+\,\sin \,3t/3$$. The inlet velocities of the main channel (Current) and the T-junction (Droplet) are kept constant, *u*_1_ = 0.01 ms^−1^ and *u*_2_ = 0.043 ms^−1^. The pulsating velocity of Injected is conditional so that it is restricted to the positive values defined in eq. (1). For negative values, velocity is set to zero.

1 $$\begin{array}{rcl}{{\boldsymbol{u}}}_{3} & = & 370[\,-\,4.325+\,{\cos }\,\{\pi ({t}+3\pi /5)\times 555\}/0.3\\  &  & +\,{\cos }\,\{2\pi ({t}+3\pi /5)\times 555\}]\,{\rm{m}}\,{{\rm{s}}}^{-1},\end{array}$$

The pulsating velocity of the injection station and the cross-junction are defined as eqs (2, 3), respectively.

2 $$\begin{array}{rcl}{{\boldsymbol{u}}}_{4} & = & 0.024[0.12+\,{\sin }\{\pi ({t}+2\pi )\times 555\}-\,{\sin }\{2\pi ({t}+2\pi )\times 555\}\\  &  & +1/3\,{\sin }\{3\pi ({t}+2\pi )\times 555\}]\,{\rm{m}}\,{{\rm{s}}}^{-1},\end{array}$$

3 $$\begin{array}{rcl}{{\boldsymbol{u}}}_{5} & = & -0.048[0.24+\,{\sin }\{\pi ({t}+2\pi )\times 555\}\\  &  & -\,{\sin }\{2\pi ({t}+2\pi )\times 555\}+1/3\,{\sin }\{3\pi ({t}+2\pi )\times 555\}]\,{\rm{m}}\,{{\rm{s}}}^{-1},\end{array}$$

Four key coefficients control the interactions among fluid phases. The appropriate selection of these coefficients guarantees the optimal performance of passive microinjection. These coefficients regulate the flow fields and are designed based on the physical conditions needed. The equation governing the pulsating flow is presented in eq. (4).

4 $${\boldsymbol{u}}=\alpha [\beta +\,{\sin }\{\gamma ({t}+{\lambda })\}-\,{\sin }\{2\gamma ({t}+{\lambda })\}+1/3\,{\sin }\{3\gamma ({t}+{\lambda })\}]\,{\rm{m}}\,{{\rm{s}}}^{-1},$$

where *γ* synchronizes the infrastructure of repeatability of the subsequent Droplets, produced at the T-junction, for the successive injection. *γ* synchronizes the injection process during the resting period. *α* is related to the momentum of flow. *β* shifts the power of pushing and pulling and adjusts the forces to move the double emulsion gently downstream preventing its collision with corners of the cross-junction. The key coefficients of the system, including *α*, *β*, *λ* are prioritized to select the optimal combination of these coefficients and achieve the highest performance of the system. First, the coefficient *γ* is used to determine the frequency of droplet formation (Droplet) derived based on the exact time of injection. The coefficient *α* and *λ* are then determined based on the position of Droplet in the injection station and magnitude of the piercing force. The coefficient *β* is finally selected to prevent collision of the double emulsion with the sharp corners of the cross-junction.

**Figure 4:**
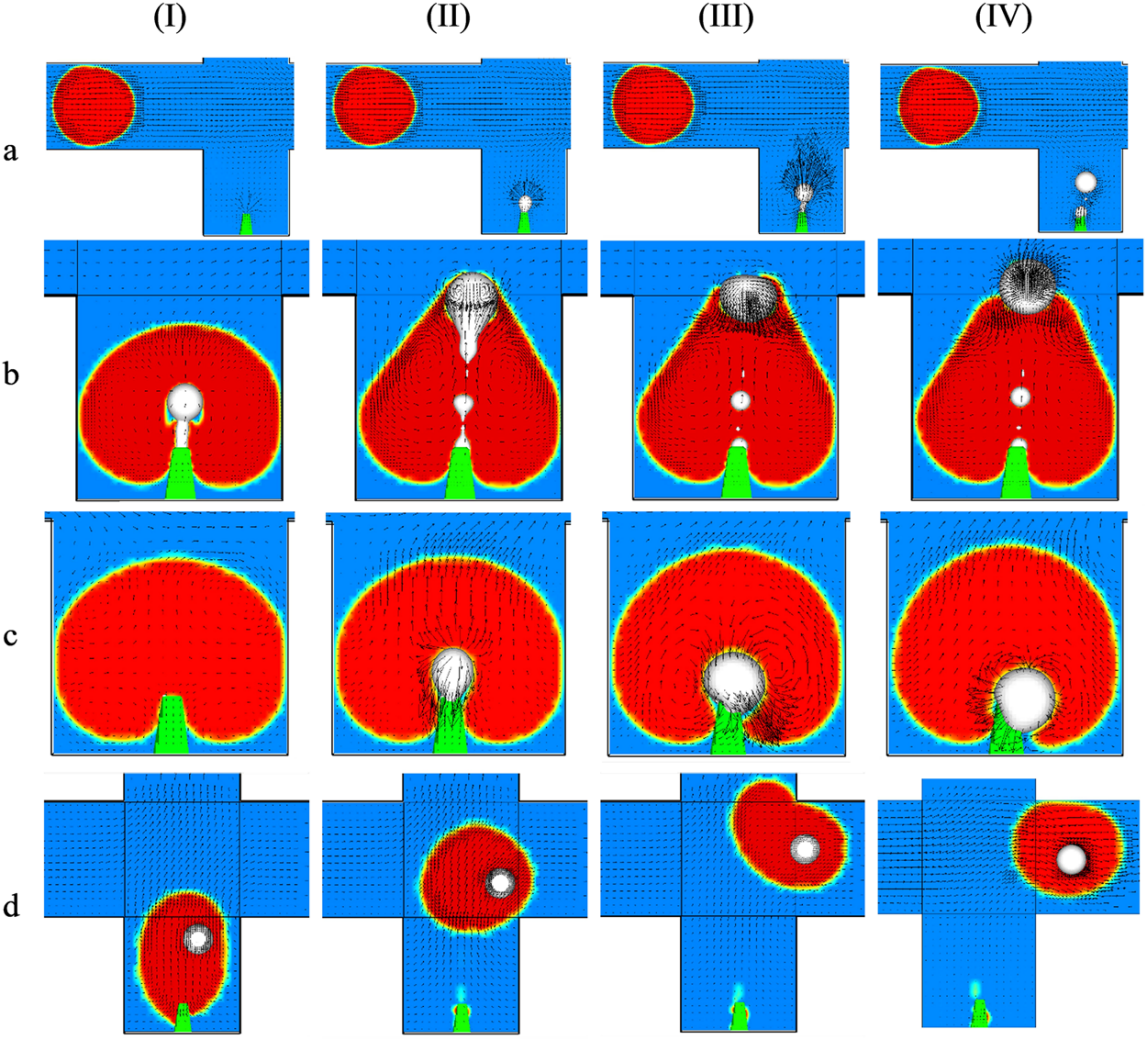
The effects of λ, α and β on the efficacy of passive microinjection. (**a**) Non-harmonized microinjection *λ* is set to $4\pi /5$. t_I_ = 0 s, t_II_ = 1.3 × 10^−5^ s, t_III_ = 2.3 ×10^−5^ s and t_IV_ = 5.4 ×10^−5^ s, (**b**) α is set to 400. t_I_ = 0 s, t_II_ = 4 × 10^−5^ s, t_III_ = 5 ×10^−5^ s and t_IV_ = 6 ×10^−5^ s, (**c**) α is changed to 300. t_I_ = 0 s, t_II_ = 6.6 × 10^−5^ s, t_III_ = 11.5 ×10^−5^ s and t_IV_ = 11.6 ×10^−5^ s, (**d**) *β* is set to zero. The shift of pushing/pulling momentum is removed. t_I_ = 0 s, t_II_ = 25 × 10^−5^ s, t_III_ = 48 ×10^−5^ s and t_IV_ = 61 ×10^−5^ s.

Several malfunction situations are studied below where the microinjection process failed because of unpunctual fluid interactions. The injection process will not be successful if the flow patterns of the pulsating flows are not regulated properly. The value *π* × 555 is set for *γ* after selecting the velocities of the main channel and the T-junction based on the needs of dripping instability^9^. Thus, it is crucial to precisely measure the period of droplet formation because the performance is highly dependent on the exact timing. Any discrepancy jeopardizes the consistency of the successive injection, and therefore some Droplets are not filled with Injected. The value *λ* needs to be determined properly to harmonize the initiation and duration of Injected with Droplet resting time at the injection station (Fig. 4a**)**. The coefficient *α* sets the momentum of each pulsating flow. If it is not appropriately set, Injected in its excessive form may either rupture Droplet or not be able to permeate Droplet (Fig. 4b,c). Finally, the coefficient *β* is related to the shift in the flow field to increase the power needed to push Droplet to the injection station respect to pulling it off the microneedle. In other words, coefficient *β* prevents the damage to double emulsions as a result of the intense collision with the sharp corners of the cross-junction (Fig. 4d). These coefficients are correlated such that successful microinjection can only be achieved via an appropriate selection of their combination. Different equations can be used for the microinjection as far as they harmonized together. For example, eq. (1) can be exchanged with eq. (5) where it is a periodic train of Gaussian pulses. More information is provided in Supplementary Information SI1.

5 $${{\boldsymbol{u}}}_{3}={\rm{A}}\times {{e}}^{-0.5[\{{\rm{B}}{\sin }^{2}(\frac{\pi }{2{\rm{B}}}({\rm{t}}-{\rm{C}}))-0.5{\rm{B}}\}/{\rm{D}}]}$$

where t is the time, e is Euler’s number, A defines the momentum power of injection, B is period of injection, C is the duration of injection and D is the shift in time or delay for each cycle of injection. Here, a similar pattern of microinjection pulse can be achieved at the microneedle if we use 3 × 10^−1^, 3.6 × 10^−3^, 1.53 × 10^−3^ and 2 × 10^−5^ for A to D, respectively.

**Figure 5:**
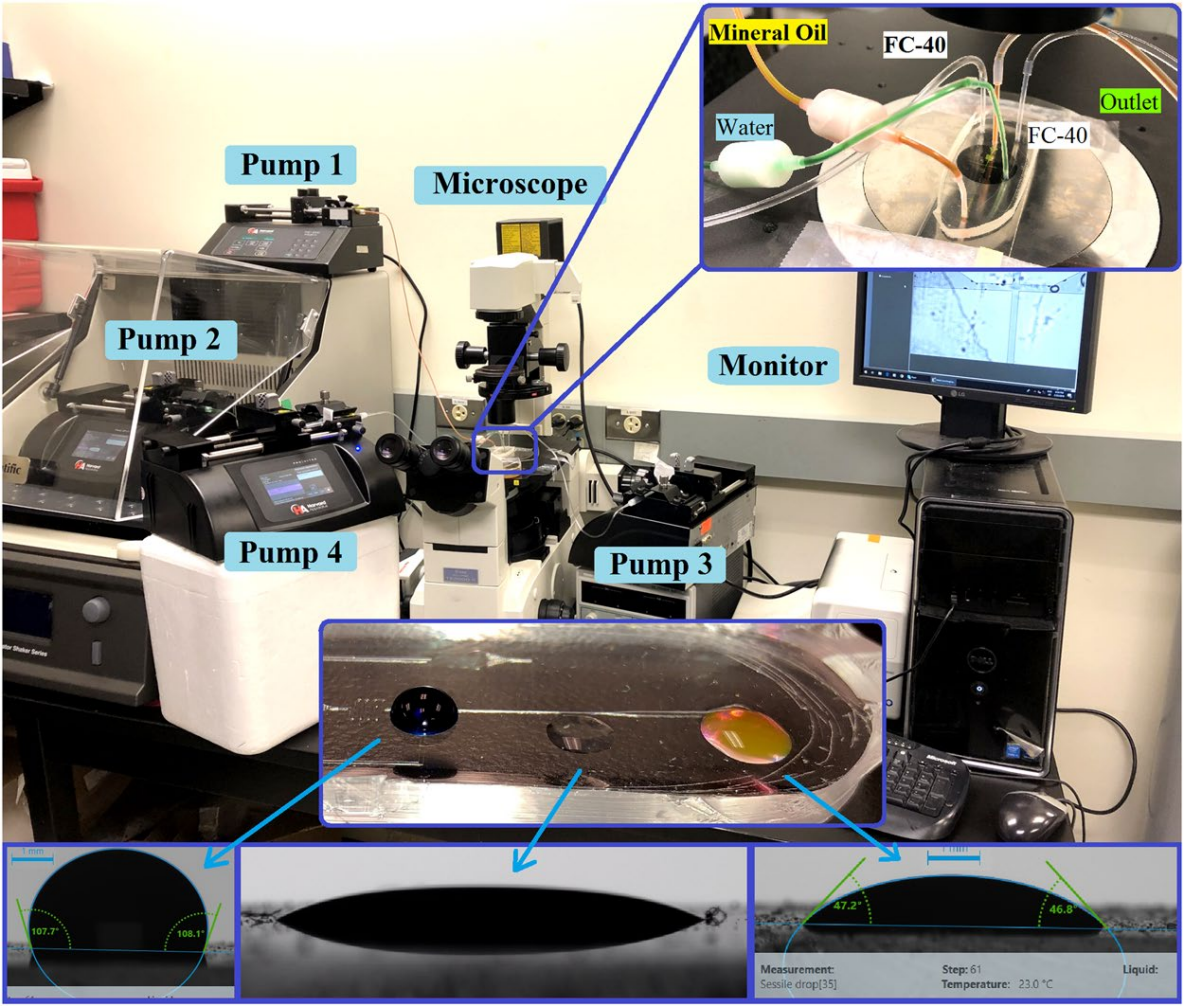
The experimental setup. The pumps are assigned with numbers of 1 to 4 which are related to mineral oil, water, the right side of cross-junction and the left side of cross-junction, respectively. The top highlighted area shows the cross-junction microchannel design used for droplet actuation. The bottom highlighted area shows static contact angle of fluids with PDMS; Left, middle and right are water, FC-40 and mineral oil, respectively.

**Figure 6:**
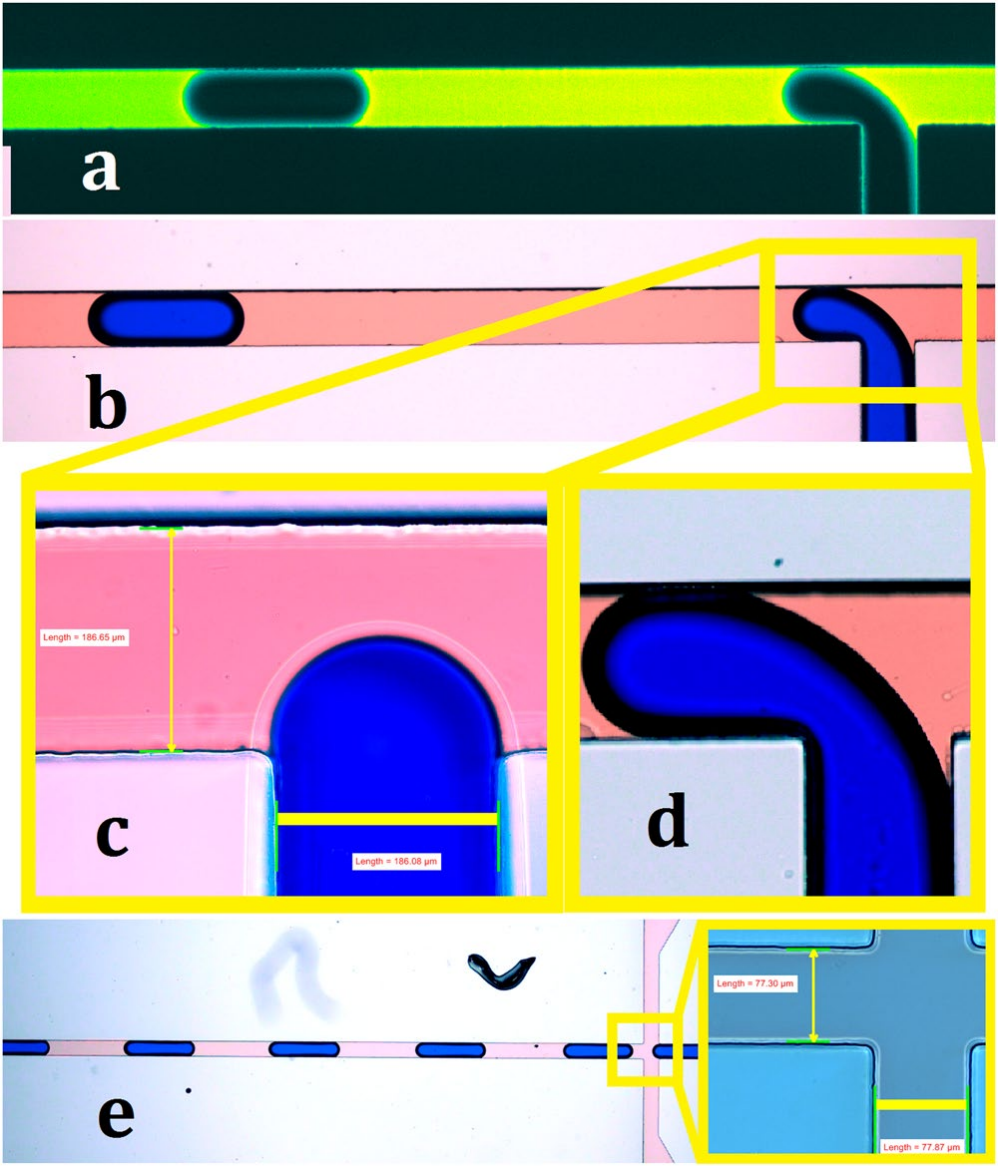
The droplet formation at T-junction and flow-focusing microchannels. (**a**) The fluorescent image that shows mineral oil is between PDMS and water droplet, (**b**) The color image of droplet formation at the T-junction. (**c**) The channel width is measured with the calibrated microscope; the scale bar is 185 $\mu m$, (**d**) The droplet curvature before detachment, (**e**) The droplet formation at the flow-focusing microchannel. The scale bar is 77 $\mu m.$

As stated above, the designed microfluid network studied in the numerical simulation has two parts of T-junction for HT generation of droplets and the microneedle station. The microneedle station itself contains two parts of uniform transport of cells/drops and delivery of the droplets/species into the cells/drops subject to pulsative flow. While the transport of cells/drops toward microinjection site behaves like flow-focusing region driven by cross-junction system, the delivery of droplets/species into pulsative-flowing drops/cells behaves like Rayleigh-Plateau instability. Therefore, to demonstrate the feasibility of three different components of our proposed microinjection system, three designs of T-junction (representing droplet generation; SV2 and SV3), flow-focusing (representing uniform cell/droplet transport; SV4) and cross-junction microchannels (representing microinjection into droplets; SV5) are experimentally tested. The setup is illustrated in Fig. 5 where three phases of water (blue, pump 2), mineral oil (red, pump 1) and FC-40 (clear, pump 3 and 4) are microscopically observed and characterized. The same physical properties for water, mineral oil and FC-40 are used for validation with numerical simulation (Table S1 and Fig. S3). The height of microchannels for all designs is set to 50 µm and the width of microchannels is shown in Fig. 6. Capillary number represents the relative effect of viscous drag forces of mineral oil versus surface tension forces between water and mineral oil.

Drop Shape Analyzer (DSA 100, Kruss) was used to measure the static contact angle of water, mineral oil and FC-40 with polydimethylsiloxane (PDMS) channel surface when air is surrounding the fluid (Fig. 3). The static contact angle is measured to be 108°, 47° and 22° for water, mineral oil and FC-40, respectively. However, the dynamic contact angle may be subject to changes when air is not the surrounding fluid. Figure 6a and SV6 show the presence of a thin oil film between the water droplet and fabricated microchannel which increases the nominal contact angle of the water droplet.

**Figure 7:**
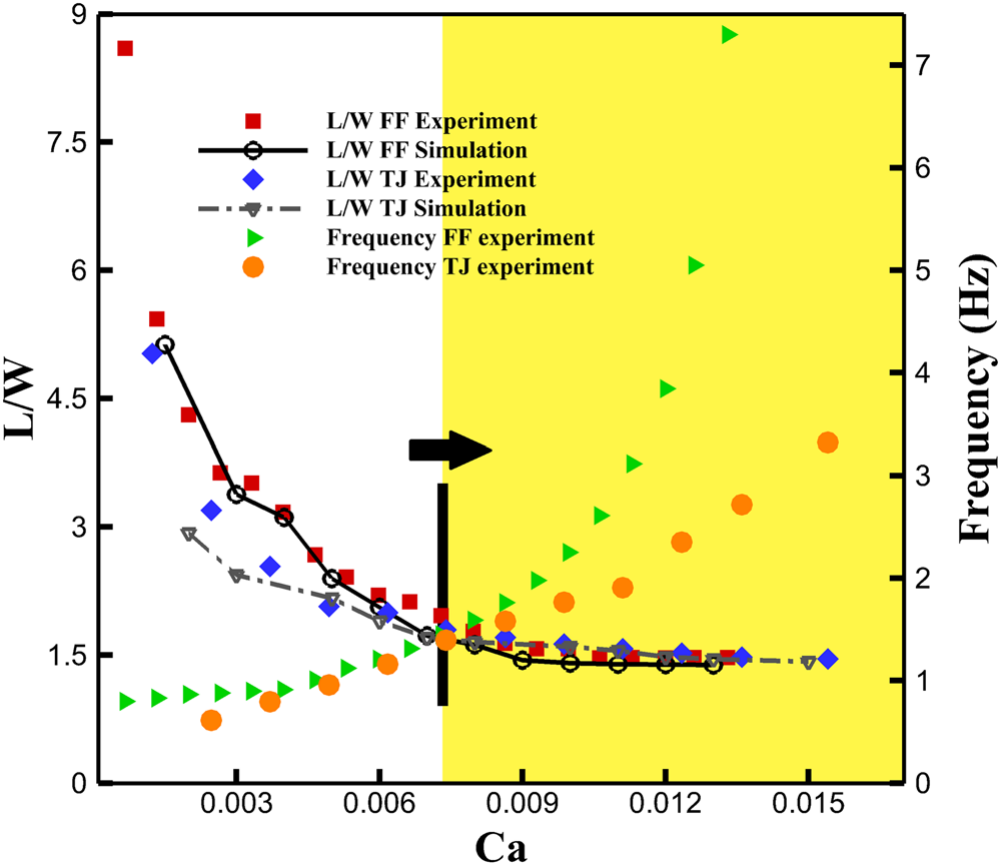
The droplet formation at the T-junction and flow-focusing microchannels. FF stands for flow-focusing and TJ stands for T-junction. The numerical simulation is validated against experimental data for droplet size. The right side is related to the frequency of droplet formation and the left side is length to width of each droplet after formation. The arrow is the boundary where the droplet size remains unchanged but the frequency of droplet generation increases considerably.

For the T-junction system, the flow rate of water at the T-junction is fixed at 40 µl/hr while it varies for mineral oil within 40–500 µl/hr. The experimental results show that increasing the flow rate of mineral oil decreases the droplet size until it reaches L/W= 1.6 at Capillary number of around 0.007 (the arrow shown in Fig. 7). For L/W higher than 1.6, the frequency of droplet formation increases without any noticeable change in droplet size that is in good agreement with numerical simulations. Similarly, the flow rate of water at the flow-focusing region is set to 10 µl/hr, while the flow rate of mineral oil varies within 10–200 µl/hr to control the water droplet size and frequency of droplet formation. Similar results are obtained for drop formation (uniform cell/drop transport) at flow-focusing microchannels where the actuation can be tuned with droplet frequency (or cell alignment frequency) to achieve the goal of microinjection. The geometry of channels defines the drop size needed for injection (representing the size of drops or cells), meanwhile increasing the carrier fluid velocity controls the frequency of drop/cell transport toward the microinjection station (Fig. 7).

**Figure 8:**
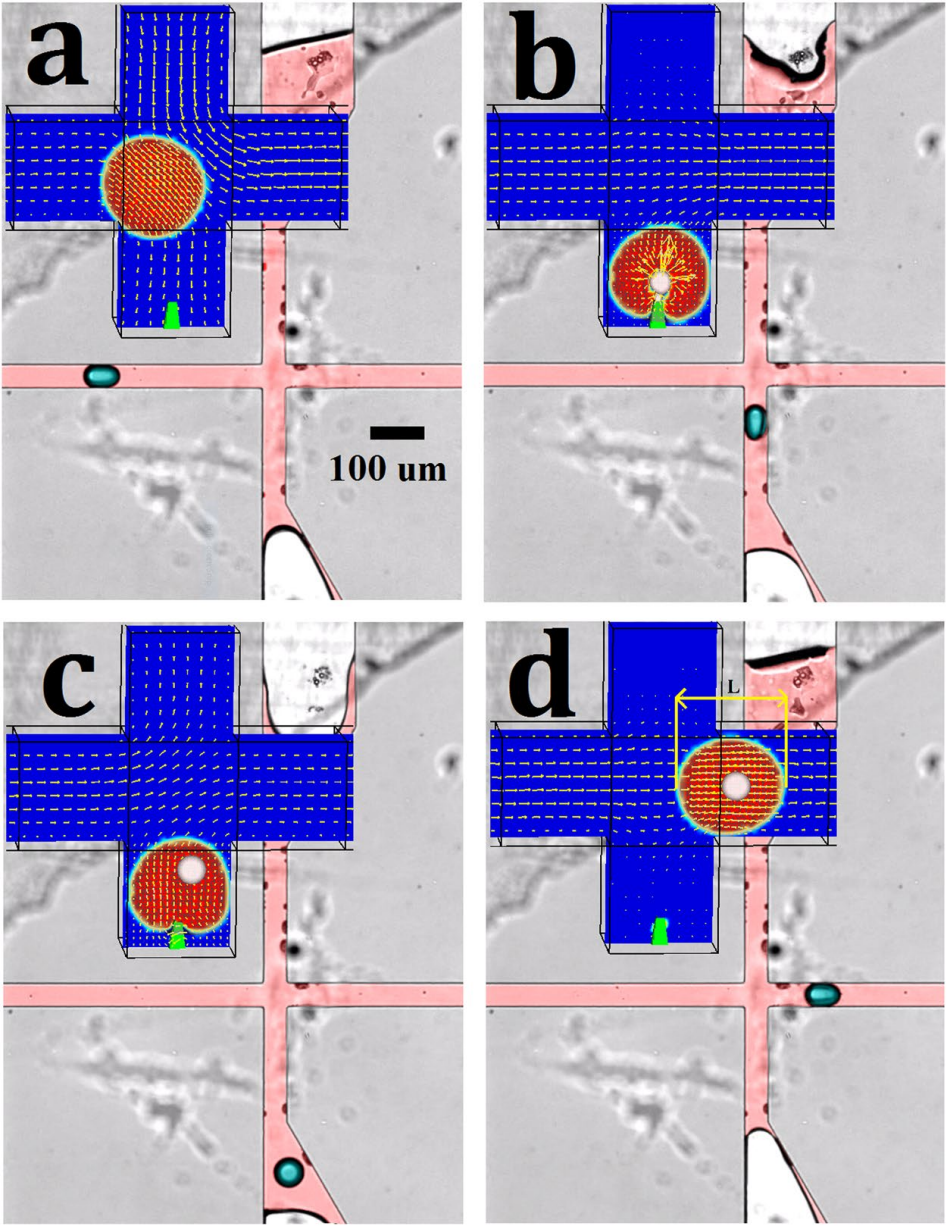
The experimental droplet actuation at the cross-junction and comparison with the numerical data. (**a**) Pushing, (**b**) Resting, (**c**) Pulling, and (**d**) Moving. The scale bar is 100 $\mu m$.

The cross-junction part is combined with the T-junction to demonstrate the feasibility of the pulsative flow of droplets (generated at upstream T-junction) at the microinjection step, similar to the numerical simulations. The fluid phases are similar to previous parts where water is Droplet phase and mineral oil is Current phase. FC-40 is used at the cross-junction to show the fluctuation near the inlets and monitor the actuation process while the entire system is tested with mineral oil and water. The fluctuation is propelled with pumps 3 and 4 where pump 3 is set to withdraw/infuse and pump 4 is set to infuse/withdraw. To set the pumps, the volume of fluid in each cycle and speed of movement are adjusted, and the frequency of cycles are controlled accordingly. For instance, in Fig. 8, the syringe diameter is 1 mm, the volume of displacement is set to 50 nl, the flow rate is set to 600 µl/hr for each cycle, the velocity of water is set to 8 µl/hr, and the velocity of mineral oil is set to 60 µl/hr. The frequency of droplet formation can reach up to 300 Hz similar to the numerical simulation. The frequency of pulsative manipulation is dependent on the frequency of actuating pumps and the pressure difference related to the size and length of tubes and channels’ hydraulic resistance. For instance, in our system, the actuation frequency of the pumps was synchronized and set three times higher than the required pulsative frequency at the cross-junction site to compensate the damping and resistance effects of tubes and channels (shown in SV5). The actuation is observed statistically stationary, and sequence of droplet actuation is the same for all droplets, which is in good agreement with numerical simulations of pulsative actuation of droplets.

## Discussion

Feasibility wise, the passive method of Adamo & Jensen^28^ with similar boundary conditions and physical aspects as our method is demonstrated. The pressure-driven microinjection methods can be implemented for both Droplets and live cells^43,44^. Also, Abate *et al*.^35^ developed an experimental setup for synchronizing multiple flows and injected a miscible phase into synchronized droplets with a period of 0.001 s. Similarly, we demonstrated experimentally the feasibility of pulsative manipulation of individual microdroplets needed for further microinjection into droplets (Fig. 8) Application wise, Villar *et al*.^43^ studied the droplets network in oil and coated them with lipid monolayers in order to assemble these droplets for the synthesis of tissues. Also, the Droplet phase after detachment behaves like particular groups of cells flowing through the channels, which demonstrates that our method with slight modification is suitable for cellular injection. This microinjection method relies on meticulous pulsating flow patterns within microfluidics. The method not only decreases the period of microinjection cycles significantly down to several milliseconds but also can create double emulsions, something that has not yet been presented via microinjection processes. The period of each injection cycle in automated cellular microinjection methods could be synchronized from 0.003 s to 10 s. The method has the potential for improving the microinjection for many applications in tissue engineering, drug discovery, cell-cell interactions, single cell analysis as well as developing new strategies for drug delivery. Our passive microinjection method is as fast as the existing methods used for the formation of double emulsions. This method particularly has the advantage of precise dosage control^35^ for Injected which makes it practical for high-resolution HT testing. The excellence of our microinjection method is also extended to the flexibility of the method in generating a variety of different immiscible inner droplet sizes (Injected) by only changing the microneedle. Several malfunction situations under which the microinjection process fails are investigated to demonstrate the essence of parameters optimization for the successful performance of this new HT microinjection system. Given the experimental success of synchronizing multiple flows within microfluidics, the proposed microinjection technique is expected to be a reliable approach for the delivery of versatile targets into single cells or droplets with increased speed and minimal errors in HT performance.

## Methods

### Numerical method

The governing incompressible Navier–Stokes eqs 6–8 involve the surface tension, variable-density flow pattern, and continuity equations for each phase. According to the continuity equation, the advection equation for the density is written by the volume fraction (eq. 9)^45^.

6 $$\rho ({\partial }_{t}{\boldsymbol{u}}+{\boldsymbol{u}}.\nabla {\boldsymbol{u}})=-\,\nabla {p}+\nabla .(2\mu {\boldsymbol{D}})+\sigma \kappa {\delta }_{s}{\boldsymbol{n}},$$

7 $${\partial }_{t}\rho +\nabla .(\rho {\boldsymbol{u}})=0,$$

8 $$\nabla {.}{\boldsymbol{u}}=0,$$

9 $${\partial }_{t}c+\nabla .(c{\boldsymbol{u}})=0,$$

where ***u***, *p*, ***D*** denote velocity vector, pressure and deformation tensor $({D}_{ij}=({\partial }_{i}{u}_{j}+{\partial }_{j}{u}_{i})/2)$, respectively. $\rho \equiv \rho ({\boldsymbol{x}},\,{\rm{t}})$ and $\mu \equiv \mu ({\boldsymbol{x}},\,{\rm{t}})$ are density and dynamic viscosity of the fluid, respectively^46^. *δ*_*S*_ is Dirac delta function which declares the fact that the surface tension coefficient *σ* is concentrated on the interface. The curvature radius of the interface is specified as *k*, and the unit vector perpendicular to the interface is denoted by ***n***^46^. The open source code Gerris is used to simulate the multiphase flows of the microinjection system. The computational method is based on the direct numerical simulation (DNS) and finite volume method (FVM) discretization of the governing equations^46^. The second-order accurate scheme is used for the spatial and temporal variables. Staggered temporal discretization is used for the volume fraction/density^45–47^. The volume of fluid (VOF) method is adopted to simulate the interfaces of three immiscible fluids and the interaction of different instabilities that detach the droplets from their reservoir. For the boundary conditions, a uniform normal velocity is applied at the inlet of the main channel (Current) (*u* = *u*_1_), and the T-junction (Droplet) (*u* = *u*_*2*_). Also, pulsative velocities are set at the inlets of microneedle (Injected, *u*_*3*_), injection station (*u*_4_), and the top part of the cross-junction (*u*_5_). The outflow boundary condition is selected for the outlet. No-slip boundary condition is selected for the microchannel walls. For the wetting property, it is assumed that the Current phase wets the entire microchannel except for the T-junction where it is wetted only by the Droplet phase. Besides, a non-wetting condition is chosen for the Injected phase.

A VOF function, *c*(***x***, *t*), is used to trace multiphase interfaces. The volume fraction field is advected by the geometrical VOF for every cell of the computational mesh^45^. The viscosity and density are defined as eqs (10,11), respectively.

10 $$\mu ({c}_{D},{c}_{I})={c}_{D}{\mu }_{D}+{c}_{I}{\mu }_{I}+(1-{c}_{I}-{c}_{D}){\mu }_{Cu},$$

11 $$\rho ({c}_{D},{c}_{I})={c}_{D}{\rho }_{D}+{c}_{I}{\rho }_{I}+(1-{c}_{I}-{c}_{D}){\rho }_{Cu},$$

where subscripts *Cu, D* and *I* are the Current, Droplet and Injected phases, respectively^2^. The cells containing multiphase interfaces are not considered as a homogenous mixture, while the phases are separated from each other with a piecewise-linear VOF scheme^10,45,47,48^. For each cell of the mesh, the surface tension force imposed on the interface represents the interaction of different phases (eq. 6). A piecewise-linear VOF method is applied for the interface reconstruction. The interface of each cell is represented by a plane defined in eq. (12)^45^.

12 $${\boldsymbol{m}}{\boldsymbol{.x}}=\alpha ,$$

where ***m*** is the local normal vector to the interface and ***x*** is the position vector. *α* is uniquely determined to ensure that the volume of fluid, maintained within the cell and placed below the plane, is equal to *c*^45^. The detailed description is explained in the references^45,46^. *C*_*D*_ = 1 and *C*_*I*_ = 1 are applied for the Droplet and Injected phases, respectively. *C*_*D*_ = *C*_*I*_ = 0 is used for complete wetting of the Current phase (the contact angle is zero). A non-wetting condition is applied to both Droplet and Injected phases where the contact angle is 180°. *C*_*D*_ = 1 for the walls of T-junction represents the wetting condition only for the Droplet phase.

Gerris code uses a semi-structure Quad/Octree spatial cells for solving the governing equations and applying the adaptive mesh refinement (AMR) technique to trace the interface^46^. AMR technique focuses on the regions of importance, for example where singularities may affect the accuracy of numerical simulation (e.g., sharp corners of the microchannel); where the gradient of physical parameters is high at the site of detachment and interface rupture; and at the gap between the droplet and microchannel walls^49^. The curvature, topology and value-based refinement are exploited concurrently to preserve numerical accuracy and robustness^2,10,47,48^ (Fig. S4).

### Geometry and grid independency

The main microchannel is simulated with constant height, width and length of *w*, *w* and 21*w*, respectively. The T-junction and cross-junction are placed at 2*w* and *7w* downstream of the main inlet, respectively (Fig. 1). The AMR technique is used with maximum three-level refinements of 6, 7 and 8, where a cell of level *n* has a resolution of ${2}^{n}$ in each coordinate^10,46^, and 0 and *n* are the refinement levels of the root cell and recursive descendant cells, respectively^2,46^. The droplet size varies about 8% with one level increase in the superlative refinement from 6 to 7 and less than 4% with one level increase in the superlative refinement from 7 to 8. Thus, the refinement level is set to 3, 4 and 7 for the main geometry, cross-junction and the microneedle (Fig. S5), respectively. The refinement level is set to 7 for the interface between Droplet and Current which ensures that the cells near the walls and Droplet are refined enough to accurately predict the shear stress exerted on Droplet. Fig. S4 shows an example of Quad/Octree AMR cells near the interfaces. The AMR technique reduced the number of total mesh points (cubic) down to approximately one million to satisfy the resolution, trace the interfaces and make an efficient grid structure for proper analysis of the interfacial dynamics. As a result, there is no need to refine the meshing near the walls because the AMR technique automatically refines the mesh when Droplet approaches the walls.

The validation of the simulation results is disseminated into the dripping instability at T-junction and Rayleigh-Plateau instability of the Droplet phase. The dripping instability at the T-junction for the formation of the Sheath is validated against the experimental data of Yeom & Lee^50^ and reported in our previous work^9^. The Rayleigh-Plateau instability of the Droplet phase arising at the expansion section is validated against the Gerris code^45^. We previously demonstrated that the simulation of the Rayleigh-Plateau instability has a good agreement with the experimental results^2^. Also, the authors previously validated the Gerris code^9^ with the droplet formation at T-junction microchannel, studied by Li *et al*.^51^ on dimensionless droplet size, and with van Steijn^52^ on velocity vectors during droplet formation process^52^.

### Experimental

The microfluidic chip was fabricated using standard soft-lithographic techniques. The photoresist master was fabricated on silicon wafers using the similar protocol as reported previously^53,54^. The photomasks were designed in AutoCAD (Autodesk) and printed as transparencies (CAD/Art Services). Briefly, SU-8-2035 Photoresist (MicroChem Corp.) was poured over four-inch silicon wafer (WRS Materials). Spin coater (Laurell Tech. Corp.) was used at the speed of 2,500 rpm to make a layer of 60 µm photoresist thickness on the silicon wafer. Following the soft baking of the resist, the wafer was exposed to UV light (AB-M Inc.) along the channel patterns on the photomask to harden the features of the microchannels. Finally, the SU8 layers were developed with SU-8 developer to remove the untouched places on the wafer. Prior to use, SU8 masters were silanized with (tridecafluoro-1,1,2,2- tetrahydrooctyl) trichlorosilane (Sigma-Aldrich) sealed over hot plate for 5 hrs at 65 °C to peel-off PDMS easily once cured. The PDMS polymer was mixed in a 10:1 ratio of base to the curing agent (Sylgard 184, Dow Corning) to make polydimethylsiloxane (PDMS) replica molds. The mixture was then degassed in a vacuum chamber for 10 min to eliminate trapped air bubbles in the PDMS sample and molded against the wafer, and then cured in the oven for 2 hrs at 75 °C. PDMS chips were removed from the molds and punched with a 1.5 mm diameter biopsy punch (Robbins Instruments) to create inlet and outlet ports. The PDMS layer and glass slide (Cover Glass, Thickness 1.5 mm, 22 mm × 40 mm, VWR international Inc.) were bonded following the oxygen plasma activation (PDC-32G, Harrick Plasma Etch). The glass substrate was cleaned with scotch tape and cleaned with isopropanol alcohol and nitrogen prior to bonding to the PDMS layer. The chips were incubated at 95 °C overnight and treated with in FC-40 (Sigma-Aldrich).

For all experiments, the Droplet phase was deionized (DI) water with 20% w/w food dye (Cole Parmer, Canada), the Carrier phase was light mineral oil (Sigma-Aldrich 330779) with 1% w/w fluorescent dye (Cole Parmer, Canada) and the Actuation phase was FC-40. Tygon Microbore tubing (1/32“ID × 1/16”OD, Cole-Parmer Canada) was connected to four programmable precision syringe pumps (Harvard Apparatus PHD2000) and used to continuously inject mineral oil, water, and FC-40 fluids into the carrier microchannels. The droplets were observed by filter-set and inverted fluorescent microscopes (Nikon EclipseTE2000-S; Nikon A1R (the color DS-Rl2 and fluorescence camera Zyla 4.2 PLUS CMOS are used), Nikon Instruments, Melville, NY). A highly sensitive monochromatic CCD camera (Moticam Pro 285A, Motic, Hong kong) was employed to capture the droplet images which were then processed by NIH ImageJ software (version 1.8.0) to determine the size, shape, and velocity of the droplets.

## Supplementary information

Video VS1

Video VS2

Video VS3

Video VS4

Video VS5

Video VS6

Supplementary information

## Data Availability

The authors declare that all data supporting the findings of this study are available within the article and its Supplementary Information files or from the corresponding author upon reasonable request.
