# Supplementary material for: Passive microinjection within high-throughput microfluidics for controlled actuation of droplets and cells
Source: Sci Rep. 2019 Apr 30;9:6723. doi: 10.1038/s41598-019-43056-2 (PMC6491429; doi:10.1038/s41598-019-43056-2)
Supplement: Supplementary file 7 — Supplementary information [file 41598_2019_43056_MOESM7_ESM.pdf]

Passive microinjection within high-throughput microfluidics for controlled actuation of droplets and cells

Milad Azarmanesh, Morteza Dejam, Pooya Azizian, Gurkan Yesiloz, Abdulmajeed A. Mohamad, Amir Sanati-Nezhad

Supplementary Information (SI)

SI1. Gaussian function is widely used in probability theory and its exponential format is defined as equation (S1).

$$f(x)=\frac{1}{\sigma\sqrt{2\pi}}\times e^{\frac{-1}{2}\left[\frac{x-\mu}{\sigma}\right]^2} \tag{S1}$$

Which μ and σ are mean value and standard deviation, respectively. Clearly by changing the variable of μ , σ and $A=\frac{1}{\sigma\sqrt{2\pi}}$, the position of the peak value, the wideness and maximum peak of the function can be customized. Given the need to a periodic function to have consecutive microinjection while the Gaussian function is not periodic, some additional mapping is needed to satisfy the problem criteria. Therefore, the time is mapped with a periodic function such as sinusoidal. Here we use $\mathrm{T}[\sin(\frac{2\pi}{4\mathrm{T}}t)]^2$ to have a periodic but positive value. The modified equation is known as periodic train of Gaussian pulses where T is period of the main function. Substituting $\mathrm{T}[\sin(\frac{2\pi}{4\mathrm{T}}t)]^2$ to the Gaussian function in equation (1) creates the ultimate function equation (2).

$$\begin{aligned} &x\rightarrow \mathrm{T}[\sin(\frac{2\pi}{4\mathrm{T}}t)]^2 \\ &\mu=\frac{\mathrm{T}}{2} \\ &f(t)=A\times e^{\frac{-1}{2}\left[\frac{\mathrm{T}[\sin(\frac{2\pi}{4\mathrm{T}}t)]^2-\frac{\mathrm{T}}{2}}{\sigma}\right]^2} \end{aligned} \tag{S2}$$

Where t is time and independent variable, and A is peak value of the function.

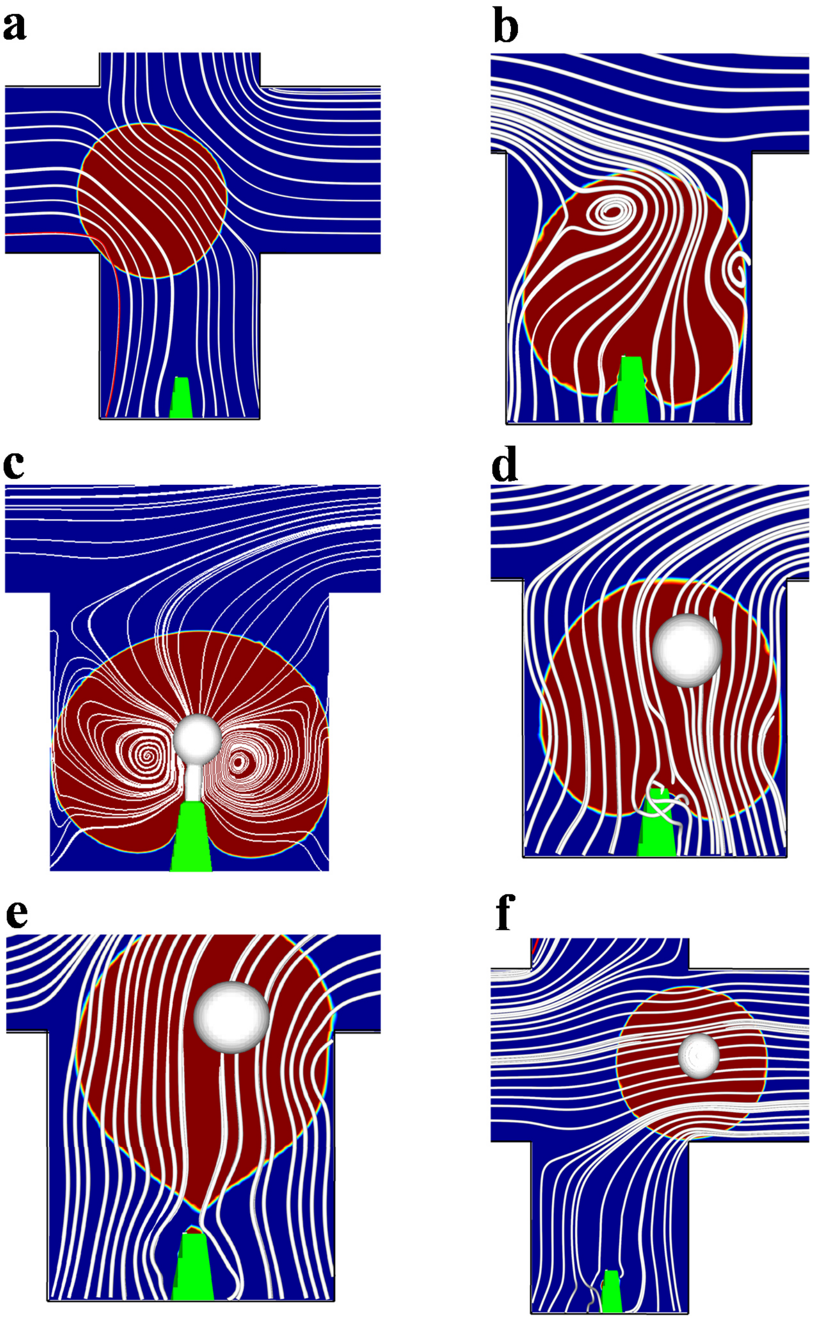

Figure S1. Streamlines of the flows in different steps of a microinjection cycle. **(a)** The pushing step moves Droplet to the injection station ($t = 6.1\times10^{-3}$ s), **(b)** The first portion of the resting step when Droplet rests over the microneedle ($t = 6.7\times10^{-3}$ s), **(c)** The second portion of the resting step when the injection initiates ($t = 6.9\times10^{-3}$ s), **(d)** The last portion of the resting step when the flow field is reversed, and the injection process is terminated. ($t = 7.1\times10^{-3}$ s), **(e)** The pulling step where the double emulsion is pulled off the microneedle ($t = 7.3\times10^{-3}$ s), and **(f)** The moving step that transfers the double emulsion to the downstream ($t = 7.8\times10^{-3}$ s).

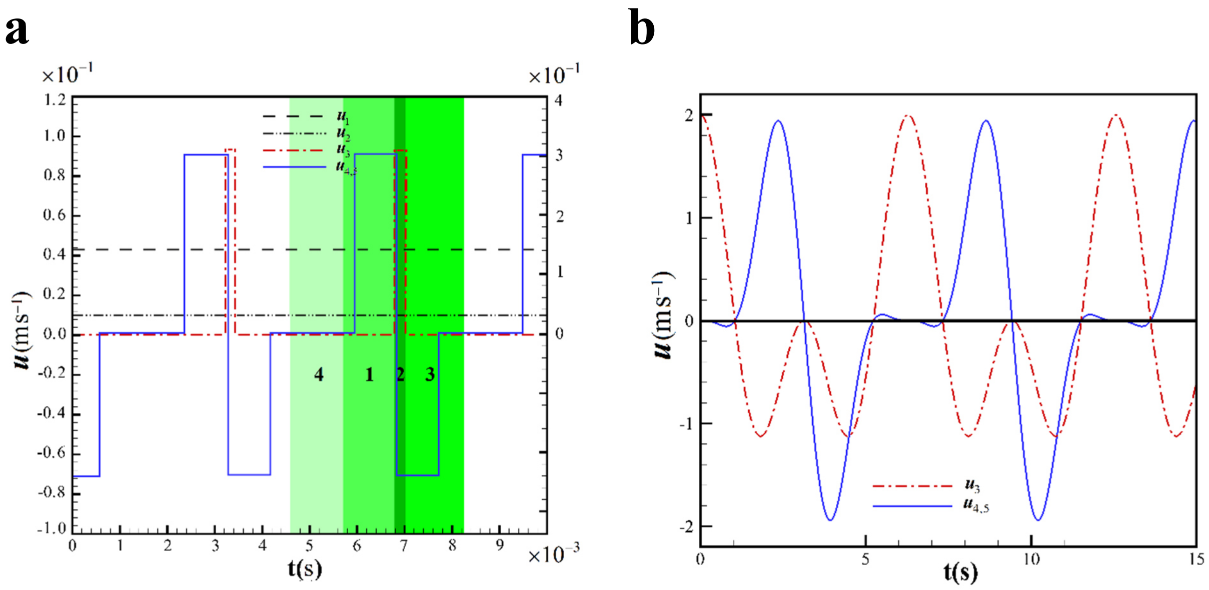

Figure S2. Preliminary pulsating flow patterns for the microinjection process. **(a)** The basic flow structure for $\mathbf{u_1}$ to $\mathbf{u_5}$, **(b)** The modified form of the flow patterns developed by Fourier series.

Table S1. Fluids properties and Interfacial tension (IFT) number. The properties are measured after adding blue food dye to water (1:5) and red fluorescent dye to mineral oil (1:100).

**Fluid at 23 °C**	**Density (kg/m^3)**	**Viscosity (Pa.s)**	**Surface tension with mineral oil (mN/m)**
Water	945	0.0007	33.77
FC-40	1855	0.0016	7.7
Mineral oil (1 CMC SPAN 20)	847	0.0307	-

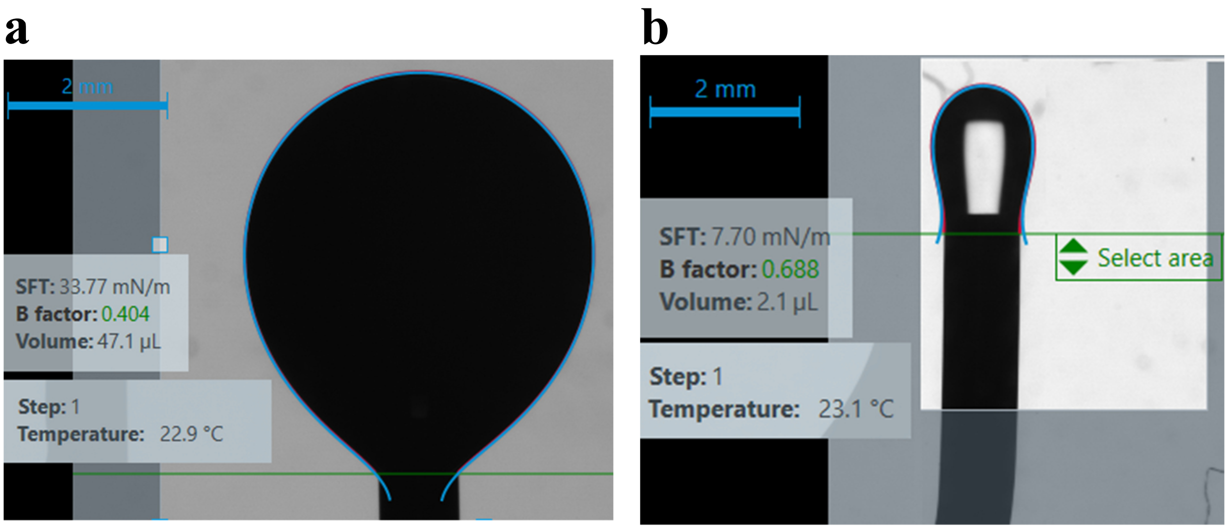

Figure S3. Interfacial tension between fluids measured by Drop Shape Analyzer (DSA 100, Kruss). Room temperature 23 ºC, **(a)** The droplet is mineral oil with 1 CMC SPAN 20 and the bath is filled with water. Interfacial tension is measured to be 33.77 mN/m, **(b)** The droplet is mineral oil with 1 CMC SPAN 20 and the bath is FC-40. Interfacial tension is measured to be 7.7 mN/m.

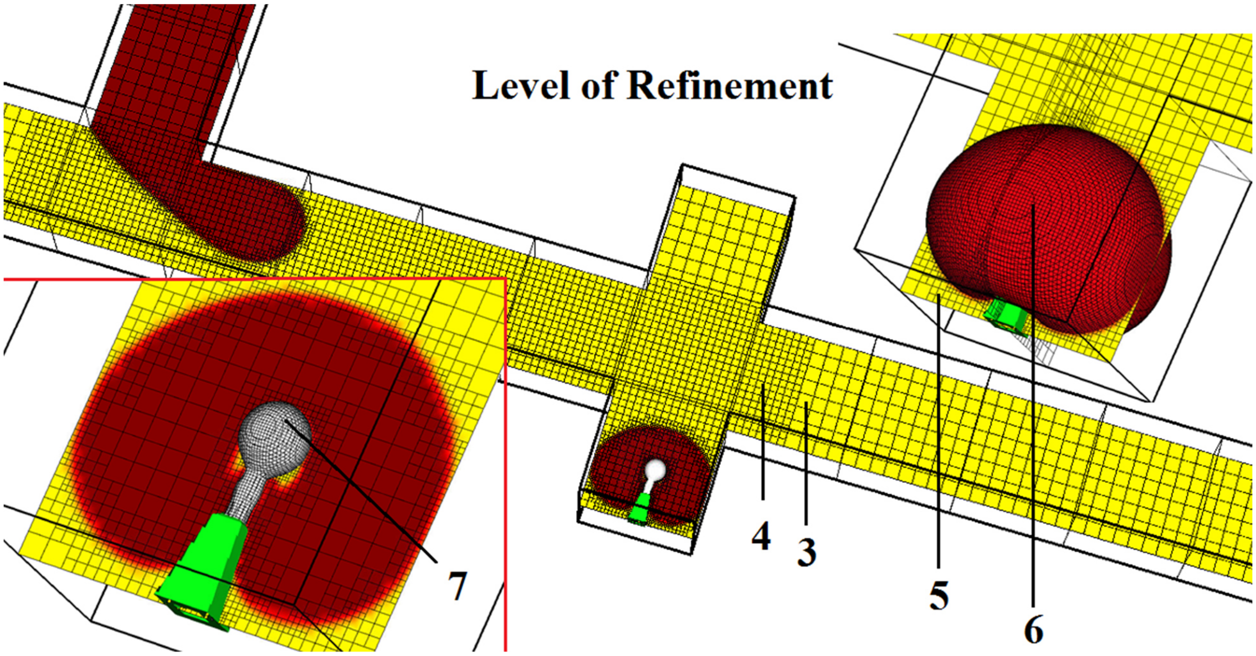

Figure S4. Discretization of the geometry by the adaptive mesh refinement (AMR) technique. The white, yellow and red colors show the Injected, Current and Droplet, respectively. Five levels of refinement are shown where 7 and 3 are related to the finest and the coarsest cells, respectively.

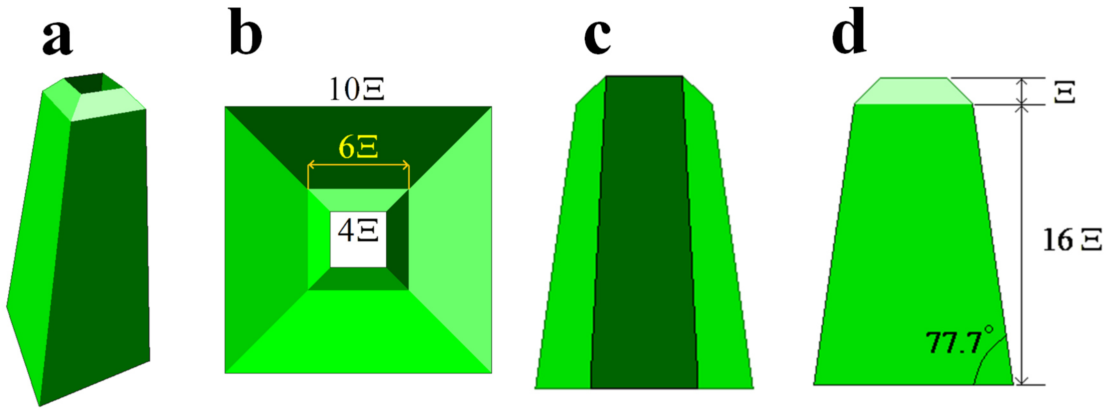

Figure S5. Schematic of the microneedle in numerical simulation, $64\,\Xi = 50\,\mu\mathrm{m}$. **a)** Isometric view, **b)** Top view, **c)** Cross section, and **d)** Side view.
